# FTLD Patient–Derived Fibroblasts Show Defective Mitochondrial Function and Accumulation of p62

**DOI:** 10.1007/s12035-021-02475-x

**Published:** 2021-07-30

**Authors:** Stina Leskelä, Dorit Hoffmann, Hannah Rostalski, Nadine Huber, Rebekka Wittrahm, Päivi Hartikainen, Ville Korhonen, Ville Leinonen, Mikko Hiltunen, Eino Solje, Anne M. Remes, Annakaisa Haapasalo

**Affiliations:** 1 A. I. Virtanen Institute for Molecular Sciences, University of Eastern Finland, Neulaniementie 2, 70211 Kuopio, Finland; 2 Institute of Biomedicine, University of Eastern Finland, Yliopistonranta 1E, 70211 Kuopio, Finland; 3 Neuro Center, Neurology, Kuopio University Hospital, 70029 Kuopio, Finland; 4 Neuro Center, Neurosurgery, Kuopio University Hospital, 70029 Kuopio, Finland; 5 Institute of Clinical Medicine – Neurosurgery, University of Eastern Finland, Yliopistonranta 1C, 70211 Kuopio, Finland; 6 Institute of Clinical Medicine – Neurology, University of Eastern Finland, Yliopistonranta 1C, 70211 Kuopio, Finland; 7 Unit of Clinical Neuroscience, Neurology, University of Oulu, P.O. Box 8000, 90014 Oulu, Finland; 8 MRC Oulu, Oulu University Hospital, P.O. Box 8000, 90014 Oulu, Finland

**Keywords:** Amyotrophic lateral sclerosis, Autophagy, C9orf72, Frontotemporal lobar degeneration, Mitochondrial function, Ubiquitin–proteasome system

## Abstract

Frontotemporal lobar degeneration (FTLD) is a clinically, genetically, and neuropathologically heterogeneous group of neurodegenerative syndromes, leading to progressive cognitive dysfunction and frontal and temporal atrophy. *C9orf72* hexanucleotide repeat expansion (C9-HRE) is the most common genetic cause of FTLD, but pathogenic mechanisms underlying FTLD are not fully understood. Here, we compared cellular features and functional properties, especially related to protein degradation pathways and mitochondrial function, of FTLD patient–derived skin fibroblasts from C9-HRE carriers and non-carriers and healthy donors. Fibroblasts from C9-HRE carriers were found to produce RNA foci, but no dipeptide repeat proteins, and they showed unchanged levels of *C9orf72* mRNA transcripts. The main protein degradation pathways, the ubiquitin–proteasome system and autophagy, did not show alterations between the fibroblasts from C9-HRE-carrying and non-carrying FTLD patients and compared to healthy controls. An increase in the number and size of p62-positive puncta was evident in fibroblasts from both C9-HRE carriers and non-carriers. In addition, several parameters of mitochondrial function, namely, basal and maximal respiration and respiration linked to ATP production, were significantly reduced in the FTLD patient–derived fibroblasts from both C9-HRE carriers and non-carriers. Our findings suggest that FTLD patient–derived fibroblasts, regardless of whether they carry the C9-HRE expansion, show unchanged proteasomal and autophagic function, but significantly impaired mitochondrial function and increased accumulation of p62 when compared to control fibroblasts. These findings suggest the possibility of utilizing FTLD patient–derived fibroblasts as a platform for biomarker discovery and testing of drugs targeted to specific cellular functions, such as mitochondrial respiration.

**Supplementary Information:**

The online version contains supplementary material available at 10.1007/s12035-021-02475-x.

## Background

Frontotemporal lobar degeneration (FTLD) is one of the most common causes of early-onset dementia in people under 65 years of age [1]. It is a clinically, genetically, and neuropathologically heterogeneous group of neurodegenerative syndromes, leading to atrophy predominantly in the frontal and temporal lobes of the brain [2] accompanied by progressive cognitive dysfunction, behavioral changes, difficulties in understanding or producing speech, and frequently neuropsychiatric symptoms. Some patients show motor symptoms resembling those observed in amyotrophic lateral sclerosis (ALS), leading to mixed disease of ALS and FTLD. Moreover, FTLD and ALS share a partially overlapping genetic and molecular pathological background [3, 4]. In addition to the sporadic forms of FTLD, approximately half of the FTLD cases can be caused by different mutations in several genes, including *GRN (Granulin)*, *MAPT (Microtubule Associated Protein Tau)*, or *C9orf72* [5–10]. The GGGGCC hexanucleotide repeat expansion in *C9orf72* (C9-HRE) is the most common genetic cause of both FTLD and ALS [9–11]. The length of the expansion can vary from tens to thousands of repeats in affected individuals. The exact pathological threshold of the C9-HRE is unclear, but fewer than 30 repeats are generally considered non-pathogenic [12].

The main suggested pathological mechanisms caused by the C9-HRE are haploinsufficiency, leading to decreased expression of the normal *C9orf72* gene products (loss-of-function), and a gain-of-toxic-function through formation and accumulation of RNA foci and dipeptide repeat (DPR) proteins (poly-GP, poly-GA, poly-GR, poly-PA, and poly-PR) generated from the expanded repeat through repeat-associated non-AUG (RAN) translation [13–16]*.* There is evidence for the contribution of both gain-of-toxic-function and loss-of-function mechanisms to the disease pathogenesis, suggesting that neurodegeneration in C9-HRE-linked FTLD and ALS could involve co-operation between the two mechanisms [17]. In addition to the abovementioned pathological hallmarks specific for C9-HRE carriers, other hallmarks such as inclusions of accumulated sequestosome 1 (p62/SQSTM1, hereafter p62) and TAR DNA-binding protein-43 (TDP-43) have been detected in the central nervous system (CNS) of FTLD and ALS patients, including patients carrying the C9-HRE [18–22].

The physiological function of C9orf72 proteins, which might be compromised by the haploinsufficiency caused by the C9-HRE, is not yet fully understood. The *C9orf7*2 gene produces three protein-coding transcript variants, which in humans are translated into two protein isoforms: the long isoform A (~50 kDa) and the short isoform B (~25 kDa) [23]*.* Isoform A, which is the main isoform expressed in neurons [16, 24], contains a differentially expressed in normal and neoplastic cells (DENN) domain, suggesting that it acts as a guanosine exchange factor for Rab-GTPases, which are important regulators of the dynamics of cellular vesicles [25, 26]. In line with this, current studies suggest that C9orf72 isoform A might be involved in the regulation of vesicular trafficking in the endosomal–lysosomal and autophagosomal–lysosomal pathways through activation of different Rab-GTPases [16, 25, 27–32]*.*

Autophagy and the ubiquitin–proteasome system (UPS) are essential pathways controlling cellular proteostasis by degrading unfolded, misfolded, and aggregated proteins. Defects in protein degradation have been implicated in the pathogenesis of several neurodegenerative diseases, including Huntington’s disease, Alzheimer’s disease, and ALS [33]. Autophagy can be induced by different stimuli, such as accumulation of misfolded or aggregated proteins or nutrient deprivation [34, 35]. In selective autophagy, ubiquitinated proteins are conjugated to adaptor molecules, such as p62, which itself is also a substrate of selective autophagy. They are then targeted to the phagophore by binding of the adaptor molecule to a membrane-bound receptor protein on the phagophore, for example, Microtubule-associated protein 1 light chain 3B (LC3B). Through elongation and subsequent fusion of the membrane endings, the phagophore forms an autophagosome, which then fuses with a lysosome, and the autolysosomal contents are degraded by lysosomal enzymes [36]. In the UPS, proteins ubiquitinated at their lysine residues are targeted to the proteasome and degraded into smaller peptides and amino acids, which can be re-utilized for protein synthesis [33]. The two pathways are suggested to be part of a single proteolytic network, co-operating in the maintenance of cellular proteostasis [33, 37, 38]. Several studies have reported that C9orf72 regulates autophagy, but results have been controversial on whether the reduced levels of C9orf72 lead to increased or decreased autophagy [16, 27–31, 39–42]**.** Moreover, it has been suggested that autophagic degradation is reduced upon loss of C9orf72 function, leading to the accumulation of DPR proteins [43]. DPR proteins, in turn, have been reported to impair protein degradation through autophagy and the UPS in C9-HRE-associated FTLD or ALS [44, 45]. These data together suggest that the C9orf72 loss-of-function might further aggravate the gain-of-toxic-function effects.

Autophagy is also intimately linked to mitochondrial quality control via a specialized form of autophagy, termed mitophagy, which eliminates damaged mitochondria [46]. The mitochondrial metabolism produces reactive oxygen species (ROS), mostly in complexes I and III of the electron transport chain (ETC) [47, 48], and mitochondrial DNA (mtDNA) might be particularly vulnerable to oxidative DNA damage [49, 50]. Even under normal conditions, reduced ATP production, accelerated production of ROS, and release of proapoptotic proteins from mitochondria may cause cellular damage. These processes are exacerbated in many neurodegenerative diseases such as Alzheimer’s disease, Parkinson’s disease, and ALS [51]. Impaired mitophagy can lead to mitochondrial dysfunction, which has been reported in both ALS and FTLD [52]. In C9-HRE carriers, mitochondrial function can also be impaired due to the expression of DPR proteins. Poly-GR proteins have been shown to bind to mitochondrial ribosomal proteins needed for the translation of mitochondrial complex subunits, leading to impaired mitochondrial function in induced pluripotent stem cell (iPSC)–derived motor neurons from C9-HRE carriers [53]. Poly-GR can also bind to ATP synthase F1 subunit alpha (ATP5FA1), a subunit of mitochondrial respiratory chain complex V, enhancing its ubiquitination and degradation and thus compromising mitochondrial function [54]. Taken together, these results suggest that mitochondrial function might be compromised due to both loss-of-function and gain-of-toxic-function mechanisms.

The research in ALS and FTLD has largely focused on pathological mechanisms in neurons. However, there are some studies demonstrating that also peripheral cells of the patients display C9-HRE-related and other pathological alterations. For example, biopsies from skeletal muscles of ALS patients carrying the C9-HRE have been reported to show both RNA foci and the poly-GA and poly-GP DPR proteins [55]. iPSC-derived skeletal myocytes from C9-HRE-carrying ALS patients also display RNA foci [56, 57] and express the poly-GR protein [57]. Moreover, RNA foci have been observed in fibroblasts from ALS patients carrying the C9-HRE [58]. In a previous study, increased levels of the autophagosome markers p62 and LC3II were present in fibroblasts derived from ALS/FTLD patients carrying the C9-HRE, suggesting impaired degradation of autophagosomes and inhibition of autophagy under stress conditions [30]. In mouse embryonic fibroblasts, *C9orf72* knockdown disrupted rapamycin-induced autophagy but, in contrast to the study on human fibroblasts, the results suggested a reduced number of autophagosomes in *C9orf72* knockdown cells [31]. Despite the discrepancies, these data link disrupted autophagy to the C9-HRE and *C9orf72* loss-of-function, and further research on their effects on autophagy is warranted. Furthermore, mitochondrial dysfunction has been observed in fibroblasts from sporadic ALS cases [59]; ALS patients carrying mutations in *VCP*, *SOD1*, or *TARDBP* (p.A382T) genes [60, 61]; and ALS and FTLD patients carrying the C9-HRE [62]. Further supporting potential mitochondrial changes in FTLD and ALS pathogenesis, RNA sequencing of iPSC-derived myocytes from ALS patients carrying the C9-HRE revealed changes in genes that regulate mitochondrial function. The myocytes were also more susceptible to oxidative stress, which might be caused by inherent mitochondrial abnormalities [57]. These data collectively suggest that also other cell types besides neurons, such as fibroblasts, may display pathological hallmarks and altered cellular function in FTLD patients. This might offer possibilities for the identification of novel biomarker candidates or using these cells as platforms for testing the effects of different therapeutic agents targeting specific cellular pathways or functions. In the present study, we have characterized fibroblasts derived from skin biopsies of FTLD patients carrying or not the C9-HRE and healthy donors. We examined the cellular pathological hallmarks related to FTLD and the C9-HRE and the functionality of the patient-derived fibroblasts, focusing especially on the protein degradation mechanisms and mitochondrial function.

## Material and Methods

### Study Subjects, Skin Biopsies, Ethical Permits, and Genotyping

Skin punch biopsies were obtained at Neuro Center, Neurology, Kuopio University Hospital, Kuopio, Finland, from six FTLD patients, three of whom were carriers of the C9-HRE and three were non-carriers, and three age- and gender-matched healthy control donors were included in the study. All the participants gave written informed consent. The study was performed according to the Declaration of Helsinki. The research in human subjects has been approved by the Research Ethics Committee of the Northern Savo Hospital District, Kuopio, Finland (ethical permits 16/2013 and 254/2015). Skin biopsy samples were pseudonymized and handled using code numbers. Studies on FTLD patient–derived skin fibroblasts have been performed with the permission 123/2016 from the Research Ethics Committee of the Northern Savo Hospital District.

The presence or absence of the C9-HRE in these individuals was confirmed from both the blood samples and the skin biopsy–derived fibroblasts by repeat-primed PCR [9]. All the three C9-HRE carriers had >60 repeats and the non-carrying FTLD patients or healthy controls all had <30 repeats.

### Culturing of Fibroblasts, Transfection, and Treatments

The skin biopsy samples were cut into pieces of approximately 1 mm^3^ in size. The pieces were then transferred to a Primaria six-well plate (# 353846; Corning) with 4–6 pieces per well and 1 ml of fibroblast medium was added. After 2, 4, and 6 days, 100, 200, and 500 μl of fresh media were added, respectively. After that, 1 ml of medium was changed every other day until the fibroblast cultures were confluent. The fibroblasts were then washed with PBS, incubated with TrypLExpress (12604013; Gibco) at 37 °C, and transferred into cell culture bottles. The fibroblasts were cultured in Iscove’s Modified Dulbecco’s Medium (IMDM, 21980032; Gibco) supplemented with 20% heat-inactivated fetal bovine serum (FBS, 10270106; Gibco), 1× MEM Non-Essential Amino Acids Solution (11140050; Thermo Fisher), 100 U/ml penicillin, and 100 μg/ml streptomycin (15140122; Thermo Fisher) (= fibroblast medium) at +37 °C and 5% CO_2_. For transfections, a total of 0.8 μg of the plasmid DNA and 2 μl Lipofectamine 2000 reagent (11668-019; Invitrogen) were used per transfection of 10,000 cells according to the manufacturer’s instructions to overexpress the GFP-tagged LC3 construct (kind gift from Prof. Kai Kaarniranta, UEF; [42]). Fresh medium was added 24 h post-transfection. To induce autophagy, 200 nM of Torin 1 (4247; Tocris) was used overnight. To assess basal autophagy, cells were treated with 300 nM bafilomycin A1 (BafA1, B1793; Sigma-Aldrich) for 6 h to block the late phase of autophagy. To block protein degradation through the UPS, 10 μM lactacystin (Enzo Life Sciences) was added overnight [27]. Dimethyl sulfoxide (DMSO, D2650; Sigma-Aldrich) was used as a vehicle control.

### Immunocytochemistry

For immunocytochemistry experiments, glass coverslips were placed in 24-well plates and coated with 0.3% gelatin for 30 min at +37 °C. Fibroblasts were plated (20,000 cells/well) and fixed after 24 h in 4% paraformaldehyde (PFA, 28908; Thermo Scientific) for 10 min at room temperature (RT). Cells were permeabilized with 0.1% Triton X-100 (X100; Sigma-Aldrich) in PBS for 10 min and blocked in 1% bovine serum albumin (BSA, A9647; Sigma-Aldrich) for 30 min (both at RT). Afterwards, the following primary antibodies were added and incubated overnight at +4 °C: anti-TDP-43 (1:100, 10782-2-AP; Proteintech), anti-phospho-TDP-43 (1:200, CAC-TIP-PTD-M01; CosmoBio), or anti-p62 (1:200; sc-28359; Santa Cruz). As secondary antibodies, goat anti-rabbit Alexa Fluor 488 (1:500, A-11008; Invitrogen) was used for TDP-43, goat anti-mouse Alexa Fluor 488 (1:500, A-11029; Invitrogen) for phospho-TDP-43, and goat anti-mouse Alexa Fluor 568 (1:500, A11004; Invitrogen) for p62. Cells were either mounted using Vectashield Vibrance antifade mounting medium containing 4′,6-diamidino-2-phenylindole (DAPI) (H-1800; Vector Laboratories) or with a 1:1 mix of mounting medium with DAPI and Vectashield Vibrance antifade mounting medium with TRITC-Phalloidin (H-1600; Vector Laboratories). Images were taken with an Olympus BX51 microscope and analyzed with ImageJ (version 1.52 p, Fiji, NIH).

For the experiments with transfected fibroblasts, glass coverslips were coated with 0.3% gelatin for 30 min at +37 °C. Fibroblasts were transfected with GFP-tagged LC3 construct. Fresh media were changed 24 h after transfection. At 30 h post-transfection, the cells were fixed in 4% PFA for 10 min at RT and mounted with Vectashield Vibrance antifade mounting medium containing DAPI. Images were taken with an LSM700 (Zeiss) confocal microscope and analyzed with ImageJ. Quantification of the LC3-positive puncta was performed as previously described [42]. First, the background was subtracted and then the image was filtered by using the blur option. Correct threshold settings were chosen to ensure that the background signal was not detected as puncta but also that the signal from the puncta was not lost. A puncta analysis tool in ImageJ was used to quantify the number of puncta in each cell.

### Fluorescence In Situ Hybridization (FISH)

FISH was performed using a protocol based on a previous publication [14], with some modifications. Cells were fixed with 4% PFA in diethyl pyrocarbonate (DEPC)–PBS, permeabilized with 0.1% Triton X-100/DEPC-PBS, and washed twice with DEPC–PBS. This was followed by incubation in hybridization buffer (10% dextran sulfate, 50% formamide, 50 mM sodium phosphate buffer (pH 7), 2× SSC) at 60 °C for 30 min. Prior to use, the locked nucleic acid (LNA) probe TYE 563-(CCCCGG)_3_ (Exiqon) and the TYE 563-(CAG)_6_ negative control probe (Exiqon) were denatured at 85 °C for 75 s and diluted to 40 nM with hybridization buffer. The hybridization of the samples with either probe was performed in a light-protected chamber at 60 °C for 16 h. Confocal images were acquired with LSM800 (Zeiss) microscope.

### TDP-43 translocation and p62 puncta analysis

Fibroblasts were stained for phospho- and total TDP-43, and p62, and with phalloidin and DAPI as described above. Microscopy images were processed using ImageJ. For (phospho)TDP-43-translocation analysis, phalloidin images were converted into binary images to depict cell bodies and measure total cell body areas [63]. DAPI images were converted into binary images to depict nuclei. Phospho- and total TDP-43 signals were quantified as sum intensities of secondary antibody fluorescence in nuclear and cytosolic areas (nuclear signal subtracted from signal within whole cell body). Sum intensities were normalized to nuclear and cytosolic areas, respectively. To determine unspecific signal intensities for each cell line, secondary antibody intensity values for nucleus and cytosol were obtained from samples stained without primary antibody. To determine the extent of TDP-43 translocation into the cytosol, a recently developed TDP-43 translocation analysis has been used. In brief, this analysis categorizes the ratio of nuclear to cytosolic TDP-43 into four categories (no, mild, moderate, severe TDP-43 translocation from nucleus to cytosol). This approach was used here to categorize both total and phospho-TDP-43 signals. For p62 puncta analysis, DAPI images were used to calculate the number cells per image. p62 images were converted into binary images and puncta with a defined size were used for further analysis. p62 signals were quantified as sum intensity of the secondary antibody and normalized to puncta area. Mean size of p62 puncta per image and mean number of p62 puncta per cell number per image were calculated. Background subtraction and thresholding was applied in that way that no puncta were detected in samples stained without primary antibody.

### Protein Extraction from Cells and Western Blotting

Proteins were extracted in lysis buffer (10 mM Tris–HCl, 2 mM EDTA, 1% SDS) supplemented with 1:100 protease and 1:100 phosphatase inhibitors (1862209 and 1862495; Thermo Scientific). Protein concentrations were measured using bicinchoninic acid assay (BCA, 23225; Thermo Scientific) and plate reader (Infinite M200; Tecan Group Ltd.). Then, 10–50 μg of protein was separated on SDS-PAGE gels (NuPAGE Novex 4-12% Bis–Tris mini or midi, NP0335 or WG1402BOX; Invitrogen) for 1 h 55 min at 100 V. Proteins were transferred on 0.2-μm polyvinylidene fluoride (PVDF) membranes (1704157; Bio-Rad) using Trans-Blot Turbo Transfer System (Bio-Rad, 25 V, 1.0 A, 30 min). After the transfer, unspecific binding sites on the membranes were blocked with 5% non-fat dry milk or bovine serum albumin (BSA A9647; Sigma-Aldrich) in 1× Tris-buffered saline with 0.1% Tween 20 (93773; Sigma-Aldrich) (TBST) for 1 h at RT. The protein bands were detected by incubating the membrane with protein-specific primary antibodies (see below) overnight at +4 °C and appropriate horseradish peroxidase-conjugated secondary antibodies (1:5000, NA934 or NA931; GE Healthcare) for 1 h at RT. The proteins were detected using enhanced chemiluminescence (ECL) detection reagents (RPN2236 or RPN2235; Amersham Biosciences, GE Healthcare) and ChemiDoc XRS+ System (Bio-Rad). The intensities of the detected protein bands were quantified with Image Lab software (6.0.1; Bio-Rad). The membrane was stripped with a stripping buffer (21063; Thermo Scientific) for 10 min at RT, after which it was washed in 1× TBST and re-probed with other antibodies. The following primary antibodies were used: anti-Fission1 (1:1000, ALX-210-1037-0100; Enzo), anti-Mitofusin (1:1000, ab57602; Abcam), anti-eIF2α (1:1000, #9722; Cell Signaling Technology), anti-phospho-eIF2α (1:1000, #3597; Cell Signaling Technology), anti-pULK1Ser757 (1:1000, #14202S; Cell Signaling Technology), anti-ULK1 (1:1000, #8054; Cell Signaling Technology), anti-C9orf72 (1:500, 22637-1-AP; Proteintech), anti-SQSTM1/p62 (#5114, 1:1000; Cell Signaling Technology), anti-LC3B (1:3000, ab51520; Abcam), anti-poly-ubiquitinated proteins (FK1, 1:1000, BML-PW8805-0500; Enzo Life Sciences), anti-TDP-43 (1:1000, 10782-2-AP; Proteintech), anti-phospho-TDP-43 (1:1000, TIP-PTD-P02; CosmoBio), anti-beta-actin (1:1000, ab8226; Abcam), and anti-GAPDH (1:5000, ab8245; Abcam).

The data are shown as median ± interquartile range or mean ± SEM. The levels of each protein were normalized to the levels of β-actin or GAPDH in the same sample and this ratio was set to 100 in (vehicle-treated) control cells. The protein levels are shown as percentage compared to those in vehicle-treated control cells (set to 100%).

### Dot Blot Analysis

For the dot blot, 1 μg of protein was added onto a nitrocellulose membrane (GE10600011; Sigma-Aldrich) and left to dry for 1 h at RT. As controls, samples from N2a cells transfected (using Lipofectamine 2000 reagent) with control (2R) and pathological (66R) GGGGCC hexanucleotide repeat expansion-containing constructs and specific constructs encoding for 100× GP, GA, GR, PA, and PR were used. Unspecific binding sites on the membranes were blocked with 5% non-fat dry milk in 1× Tris-buffered saline with 0.1% Tween 20 (Sigma-Aldrich) (TBST) for 1 h at RT. The proteins were detected by incubating the membrane with protein-specific primary antibodies (see below) for 30 min at RT and appropriate horseradish peroxidase-conjugated secondary antibodies (1:5000, NA935, NA934, or NA931; GE Healthcare) for 30 min at RT. The proteins were detected using enhanced chemiluminescence (ECL) detection reagent (RPN2236; Amersham Biosciences, GE Healthcare) and ChemiDoc XRS+ System (Bio-Rad). The following primary antibodies were used: anti-poly-GA (1:1000, MABN889; EMD Millipore), anti-poly-GP (1:1000, ABN455; EMD Millipore), anti-poly-GR (1:1000, MABN778; EMD Millipore), anti-poly-PR (1:1000, 23979-1-AP; Proteintech), and anti-poly-PA (1:1000, ABN1356; EMD Millipore).

### Proteasomal Activity Measurement

Proteasomal chymotrypsin-like activity was measured with a UBPBio kit (J4110) or Abcam proteasomal activity kit (ab107921) according to the kit instructions. Briefly, proteins, including proteasomes, were extracted from all fibroblast lines using a 0.5% NP-40 (Sigma-Aldrich) lysis buffer (prepared in distilled water) and centrifugation at 16,000×*g* for 20 min at +4 °C. The protein concentrations were measured using a Pierce BCA Protein Assay Kit and adjusted to same in all samples. In two separate wells, 10 μl of each protein lysate was incubated with the proteasomal substrate Succ-LLVY-AMC without or with the proteasomal inhibitor MG-132 (negative control). The resulting fluorescence, i.e., proteasomal activity, was measured at excitation/emission wavelength of 360 nm/460 nm, respectively, with an Infinite M200 (Tecan) plate reader. To specifically acquire the activity of only the proteasomes, excluding the activity of other proteases present in the sample, the fluorescence value of the corresponding MG-132-treated sample was subtracted from the total fluorescence value in the sample without MG-132 treatment. The values were further normalized to the protein concentration of each sample and shown as percentage of control fibroblast samples (set to 100%).

### Mitochondrial Function Assay

For the experiments on mitochondrial function, fibroblasts were plated (5000 cells/well) in an uncoated Seahorse XF96 Cell Culture Microplate (101085-004; Agilent) with 8 wells per cell line in each experiment. The Cell Mito Stress Test was performed 48 h after plating using assay parameters provided by Agilent. On the day of the experiment, medium was changed to Seahorse XF DMEM medium (103575-100; Agilent) supplemented with 10 mM Seahorse XF glucose solution, 2 mM Seahorse XF L-glutamine solution, and 1 mM Seahorse XF pyruvate solution (103577-100, 103579-100, and 103578-100, all from Agilent) and cells were kept in a CO_2_-free incubator for 1 h prior to starting the Cell Mito Stress Test. For the experiments, the following final concentrations of ETC modulators were used: carbonyl cyanide-4-(trifluoromethoxy)phenylhydrazone (FCCP) 2 μM, oligomycin 1 μM, and a mixture of antimycin A 1 μM and rotenone 1 μM (C2920, 75351, A8674, and R8875, all from Sigma-Aldrich). Changes in oxygen consumption rate (OCR) in response to injections were detected with Seahorse XFe96 analyzer (Agilent). In the Seahorse Cell Mito Stress Test, after measuring basal respiration, oligomycin, which blocks complex V (ATP synthase), is added. The subsequent decrease in OCR is linked to cellular ATP production. The uncoupling agent FCCP collapses the proton gradient, leading to uninhibited electron flow through the ETC and oxygen consumption by complex IV reaches the maximum. With the OCR following FCCP injection, the spare capacity can be calculated, which is a measure of the cell’s ability to respond to an increased energy demand. The injection of rotenone and antimycin A blocks complexes I and III, respectively, and shuts down mitochondrial respiration completely, allowing the calculation of non-mitochondrial respiration driven by processes outside the mitochondria [64]. For normalization of the data, cells were stained with Vybrant DyeCycle Green Stain (5 μM, V35004; Thermo Fisher) after completing the Cell Mito Stress Test and microscopy images were acquired with 4× objective from brightfield and green fluorescence channel using IncuCyte S3 (Essen BioScience). IncuCyte software (v2019B) was used to count the number of cells per well. Mitochondrial parameters were calculated using the Wave 2.6.0 software (Agilent), and results were normalized to the number of cells counted per well.

### TaqMan Assay for *C9orf72* Transcripts

The RNA from fibroblast lines was extracted with RNA extraction kit (11828665001; Roche) and concentrations were measured with NanoDrop One (Thermo Scientific). A total of 1000 ng of extracted RNA was reverse transcribed into cDNA using random hexamer primers (Roche). *C9orf72* total (both isoforms A and B) and isoform A-specific RNA transcript levels were assessed in triplicates with TaqMan assays (Hs00945132_m1 for transcript variants 2 and 3 (= isoform A) and Hs00376619_m1 for transcript variants 1, 2, and 3 (= total *C9orf72*), both ThermoFisher) and TaqMan Fast Advances Master Mix using LightCycler 480 II (Roche). Final results were obtained by normalizing the Ct values to those of β-actin and using the −ΔΔCt method to determine the expression levels compared to the controls.

### Statistical Analyses and Presentation of Data

The data are shown either as median ± interquartile range or mean ± SEM, depending on their distribution, as indicated in the figure legends. Statistical analyses were performed using GraphPad Prism5 (version 8.3.1). Shapiro–Wilk test was used to test if data points were normally distributed. For data with more than two groups and no additional variables (i.e.*,* no treatment with Torin 1, Lactacystin, or Bafilomycin A1) either one-way ANOVA (normally distributed data) or Kruskal–Wallis test (not normally distributed data) was performed. If a significant difference was observed in the initial ANOVA, this was followed by either Tukey’s or Sidak’s multiple comparison test (for normally distributed data) or Dunn’s multiple comparison test (for not normally distributed data). *P* values ≤0.05 were considered statistically significant and only *p* values that were significant in the post hoc tests are indicated in the graphs. For data with more than two groups and an additional variable (i.e., treatment with treatment with Torin 1, Lactacystin, or Bafilomycin A1), two-way ANOVA was performed (with or without transformation of the data). If a significant difference was observed in the initial ANOVA, this was followed by Tukey’s multiple comparison test and only *p* values that were significant in the post hoc tests are indicated in the graphs.

Graphs were drawn using the GraphPad Prism software (version 8.3.1). For Western blot, samples from independent experiments were considered biological replicates. For proteasomal activity, cells of the same passage plated in separate wells before the measurements were considered biological replicates. In the Seahorse assay, results from plating of different passages were considered biological replicates. For immunofluorescence data quantification (p62, TDP-43, and phospho-TDP-43), individual pictures, each containing several cells, taken from the same coverslip were considered biological replicates. For LC3-positive puncta, each analyzed cell was considered a biological replicate. The number of *n* indicated in the figure legends describes the number of biological replicates according to the definitions above.

## Results

### C9-HRE Carrier Fibroblasts Do Not Show Altered *C9orf72* mRNA or Protein Levels nor Express DPR Proteins But Display RNA Foci

**Fig. 1:**
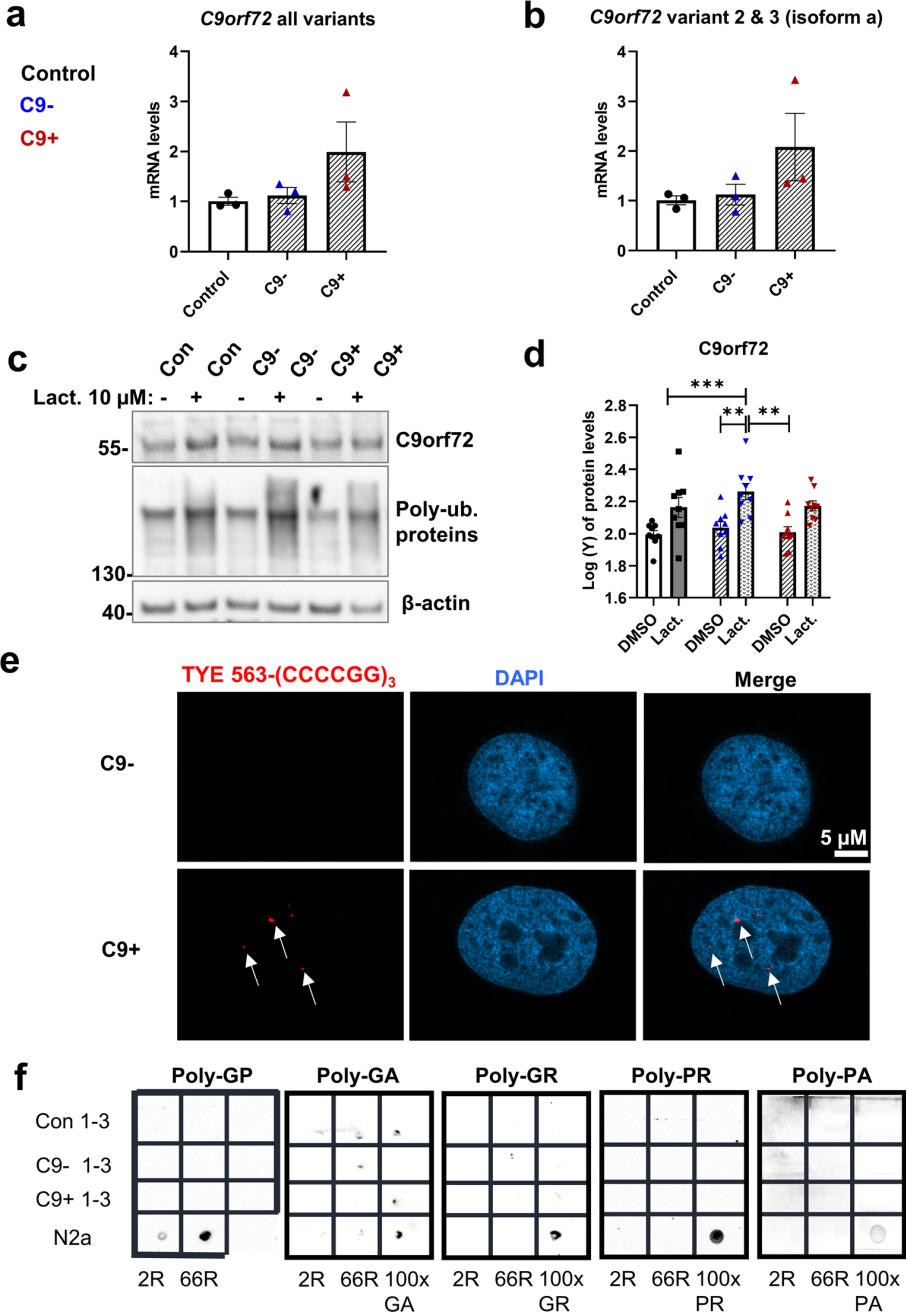
Fibroblasts of C9-HRE carriers show unaltered *C9orf72* levels, express RNA foci, but do not display DPR proteins. **a** TaqMan assay from fibroblast RNA for *C9orf72* all variants. **b** TaqMan assay from fibroblast RNA for isoform A specific transcript levels. For (**a**) and (**b**), data are shown as mean of data points in one biological replicate ± SEM. One-way ANOVA followed by Sidak’s multiple comparison test was performed. Only *p* values that were significant in the post hoc test are indicated in the graph. **c** A representative Western blot of the total protein lysates of fibroblasts from a control subject (Con), FTLD patient without the C9-HRE (C9−), and FTLD patient with the C9-HRE (C9+). Cells were treated with 10 μM lactacystin (Lact.) overnight to block protein degradation through UPS. Poly-ubiquitinated proteins (poly-ub. proteins) accumulated similarly in all the fibroblast lines treated with lactacystin. DMSO was used as a vehicle. **d** Quantification of the C9orf72 levels from the Western blot images. Data are shown as the mean of three biological replicates ± SEM. Data were transformed to achieve normality and two-way ANOVA followed by Tukey’s multiple comparison test was performed. *p ≤ 0.05, **p ≤ 0.01. Only *p* values that were significant in the post hoc test are indicated in the graph. **e** A Representative image of RNA foci (red) in the fibroblasts of a C9-HRE carrier (lower images). A C9-HRE non-carrier does not show any RNA foci (upper images). DAPI (blue) was used to stain the nuclei. **f** Dot blot images of the total protein lysates of fibroblasts from controls, FTLD patients without the C9-HRE (C9− 1–3), and FTLD patients with the C9-HRE (C9+ 1–3). Lysates from N2a cells transfected with 2R plasmid were used as negative control and lysates from N2a cells transfected with 66R plasmid (positive control for Poly-GP) or plasmids encoding the individual 100× DPR proteins (positive control for poly-GA, poly-GR, poly-PR, and poly-PA) were used as positive controls

Previous studies have suggested that the *C9orf72* haploinsufficiency, leading to decreased *C9orf72* mRNA and protein levels, associates with C9-HRE-related pathogenesis. To assess whether FTLD patient–derived fibroblasts from C9-HRE carriers show alterations in *C9orf72* mRNA levels compared to fibroblasts from FTLD patients not carrying the C9-HRE or control subjects, total and isoform A-specific *C9orf72* mRNA levels were detected using qPCR. Based on this analysis, we did not observe decreased *C9orf72* transcript levels in the fibroblasts of C9-HRE carriers in comparison to non-carriers or controls. In fact, fibroblasts from one C9-HRE carrier even showed increased *C9orf72* transcript levels (Fig. 1a, b).

To study possible differences in the C9orf72 expression at the protein level between C9-HRE carriers, non-carriers, and controls, protein samples were extracted. Some of the cells were treated with the proteasomal inhibitor lactacystin to assess the possible regulation of C9orf72 levels via UPS. Similar to the results at the mRNA level, we did not observe any differences in C9orf72 protein levels based on Western blot analysis between the C9-HRE carriers, non-carriers, or controls (Fig. 1c, d). A significant increase in C9orf72 levels after lactacystin treatment was detected in fibroblasts without the C9-HRE, with a similar trend showing in healthy controls (*p* = 0.07) and fibroblasts with the C9-HRE (*p* = 0.08) (Fig. 1c, d), which might suggest proteasomal regulation of C9orf72 protein levels in the fibroblasts in a similar manner to our previous studies in mouse neuronal cells overexpressing the C9orf72 isoform A [42]. These findings altogether suggest that the C9-HRE carrier fibroblasts do not show evidence for *C9orf72* haploinsufficiency at the mRNA or protein level.

Several previous studies have indicated the presence of RNA foci in fibroblasts, cortex, spinal cord, white blood cells, and iPSC-derived skeletal myocytes of C9-HRE-carriers, but whether the fibroblasts express the DPR proteins, the other key C9-HRE gain-of-toxic-function-associated pathological hallmark, is not clear [23, 57, 58, 65]. FISH analysis indicated that the fibroblasts from the C9-HRE carriers specifically displayed RNA foci, whereas the fibroblasts from non-carriers did not (Fig. 1e), similarly to previous studies [65–67]. No foci were detected when the fibroblasts from C9-HRE carriers and non-carriers were probed with the negative control probe detecting GAC repeats (Fig. S1), further indicating the specificity of the analysis. The expression of the poly-GP, poly-GA, poly-GR, poly-PR, and poly-PA DPR proteins in the patient fibroblasts was assessed by dot blot analysis similarly to a previous study in skeletal muscle samples [55]. Samples from N2a mouse neuroblastoma cells transfected with a plasmid encoding 66 GGGGCC expanded repeats (66R, [68] for poly-GP) or plasmids encoding the individual DPR proteins (100×, [69] for poly-GA, poly-GR, poly-PR, and poly-PA) were used as positive controls in the analyses. This analysis indicated that the C9-HRE-carrying fibroblasts did not express detectable levels of the DPR proteins (Fig. 1f).

### Fibroblasts from FTLD Patients Do Not Show Alterations in Basal Autophagy, But Display Increased p62 Puncta

**Fig. 2:**
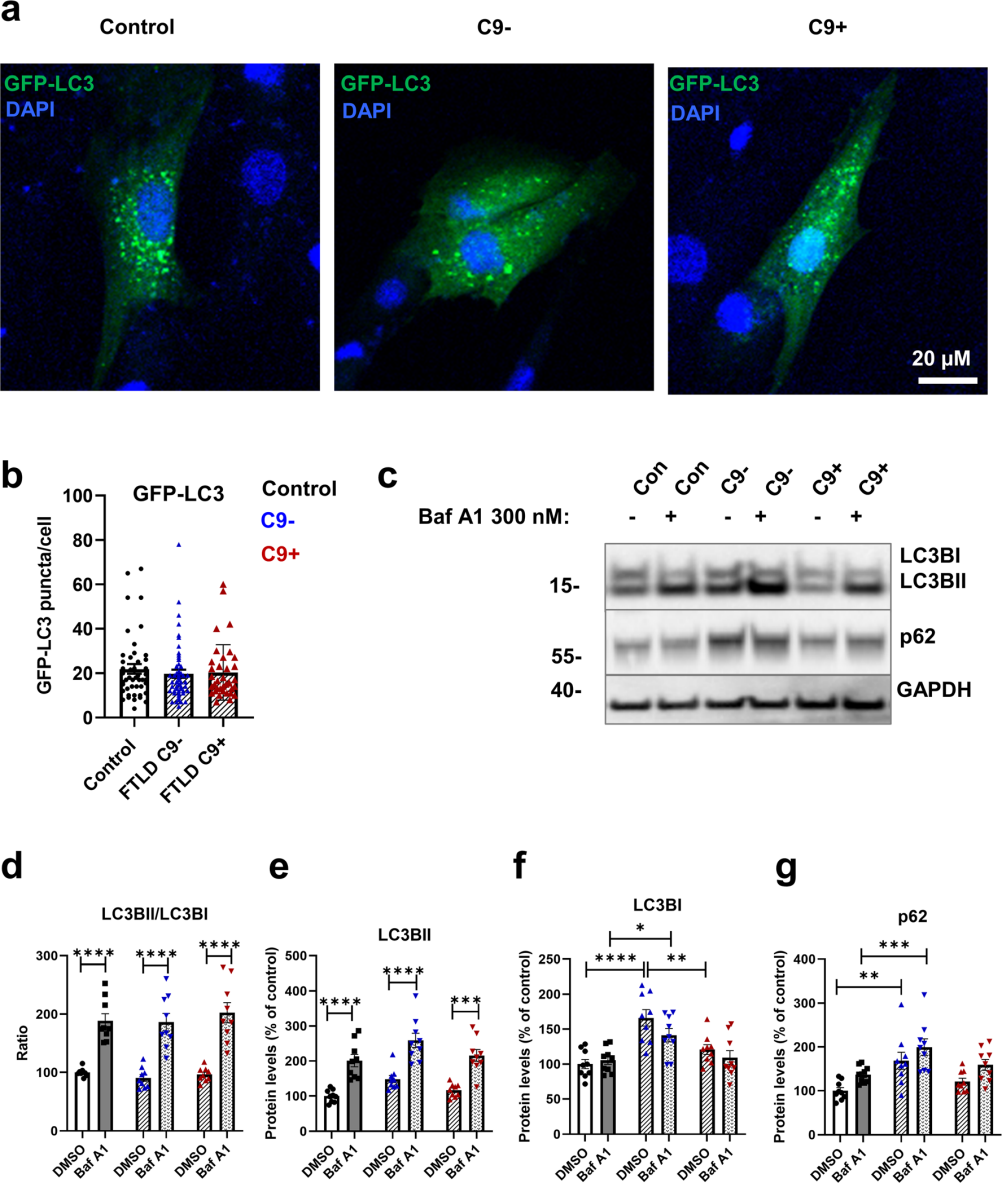
Fibroblasts from FTLD patients do not show alterations in autophagy. **a** Representative fluorescent microscope images of the control, FTLD without (C9-), and FTLD with the C9-HRE (C9+) fibroblasts transfected with GFP-LC3 plasmid. **b** Quantification of the GFP-LC3 puncta. Data are shown as the median ± interquartile range and Kruskal–Wallis followed by Dunn’s multiple comparison test was performed. Only *p* values that were significant in the post hoc test are indicated in the graph. Number of cells analyzed: *n* = 46 control, *n* = 87 FTLD. Each datapoint (=cell) represents a biological replicate. **c** Representative Western blot images from LC3BI and II, p62 and GAPDH from fibroblast cell lysates. Cells were treated with 300 nM bafilomycin A1 (BafA1) for 6 h to block the fusion of autophagosomes with lysosomes. DMSO was used as a vehicle. **d** Ratio of LC3BII/I. **e** Quantification of LC3BII. **f** Quantification of LC3BI. **g** Quantification of p62. Data are shown as the mean of three biological replicates ± SEM and two-way ANOVA followed by Tukey’s multiple comparison test was performed (**d**–**g**). Only *p* values that were significant in the post hoc test are indicated in the graphs. *n* = 9 control, *n* = 9 FTLD with C9-HRE, and *n* = 9 FTLD without C9-HRE. *p ≤ 0.05, **p ≤ 0.01, ***p ≤ 0.001, ****p ≤ 0.0001

Defects in autophagy are suggested to contribute to the pathogenesis of FTLD and ALS [33]. During autophagy induction, phosphatidylethanolamine (PE) is conjugated with cytosolic LC3BI to form a membrane-bound lipidated LC3BII. Consequently, increased levels of LC3BII or increased LC3BII/LC3BI ratio can be used as a marker of autophagy induction and the number of autophagosomes present in the cells [35]. Accumulation of p62, a known autophagy receptor and substrate, has been observed in the brain of C9-HRE carriers and could indicate compromised autophagy [18]. To assess basal autophagy, the control and FTLD patient–derived fibroblasts were treated with BafA1 to block the fusion of autophagosomes with lysosomes and, thus, the late phases of the autophagosomal degradation pathway [35, 42, 70]. Subsequently, the protein levels of LC3BI, LC3BII, and p62 were analyzed using Western blot (Fig. 2c–g). Also, the number of GFP-LC3-positive puncta in the fibroblasts was quantified from immunofluorescence images (Fig. 2a). Slightly increased LC3BI protein levels in FTLD patient fibroblasts without the C9-HRE were observed compared to fibroblasts with the C9-HRE and healthy controls in vehicle treatment (DMSO) (Fig. 2c, f), but no significant differences in the protein levels of LCBII (Fig. 2c, e) or the ratio of LC3BII/LC3BI (Fig. 2c, d) were detected between the fibroblasts from controls and FTLD patient fibroblasts with and without the C9-HRE. Treatment with BafA1 increased the LC3BII levels and thus the ratio of LC3BII/LC3BI to a similar extent in all fibroblasts, suggesting that basal autophagy was not altered in any of the fibroblasts. The number of GFP-LC3-positive puncta was also unchanged (Fig. 2b), further indicating that autophagosome formation and basal autophagic flux in FTLD patient fibroblasts were not affected.

**Fig. 3:**
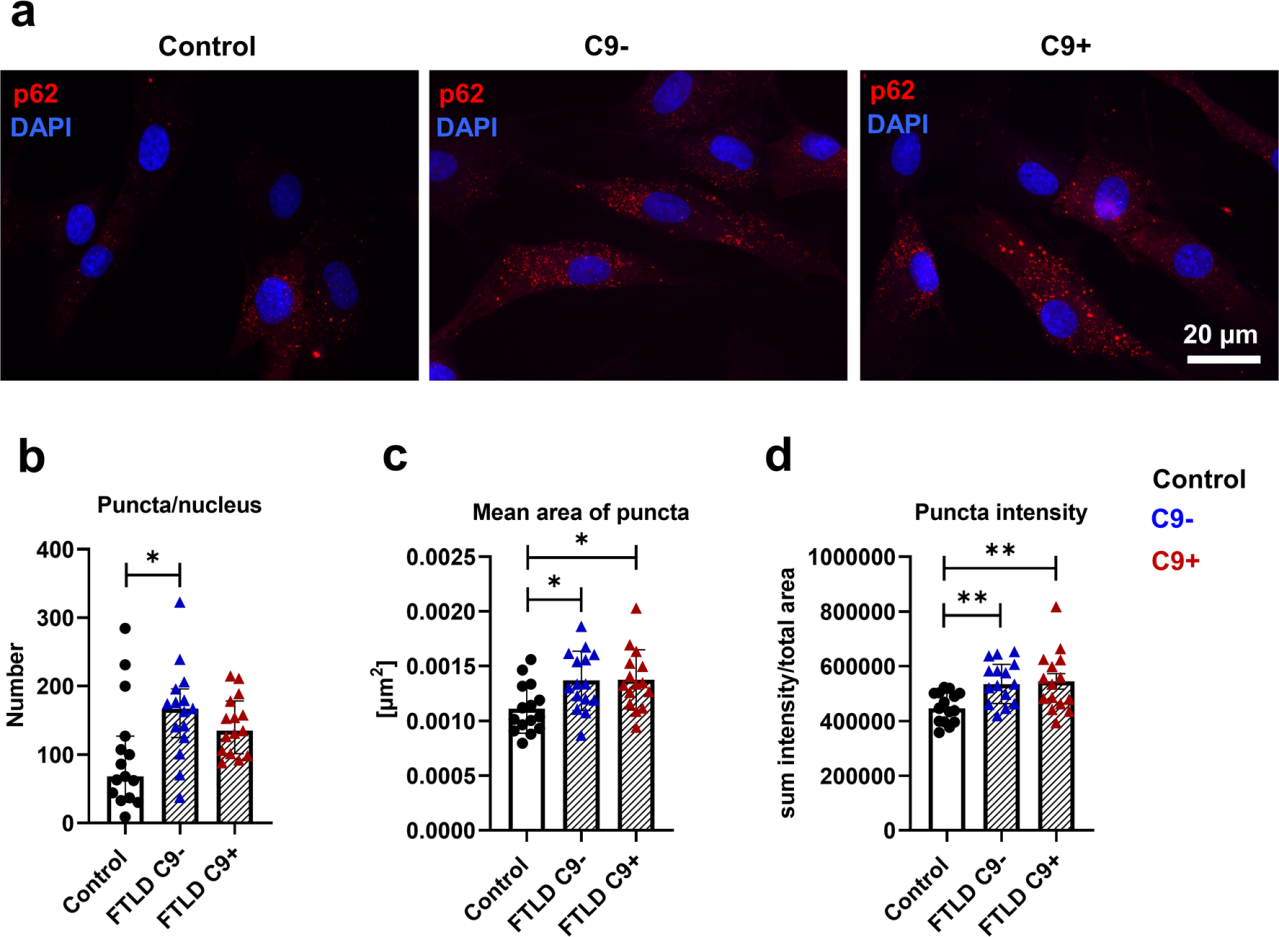
Number, size, and intensity of p62 puncta are increased in FTLD patient–derived fibroblasts. **a** Representative fluorescence microscopy images of staining with anti-p62 antibody (red) in fibroblasts of control, FTLD patient without (C9-), and FTLD patient with the C9-HRE (C9+). Nuclei were stained with DAPI (blue). **b** Quantification of number of p62 puncta. **c** Quantification of mean area of p62 puncta. **d** Quantification of intensity of p62 puncta. Data are shown as mean ± SEM and one-way ANOVA followed by Sidak’s multiple comparison test was performed (**a**, **b**) or median ± interquartile range and Kruskal–Wallis followed by Dunn’s multiple comparison test was performed (**c**, **d**). Only *p* values that were significant in the post hoc test are indicated in the graphs. Number of images analyzed *n* = 15 for control, *n* = 15 FTLD with C9-HRE, and *n* = 15 FTLD without C9-HRE. Data were obtained from one experiment and each datapoint represents a biological replicate. **p ≤ 0.01, ***p ≤ 0.001, ***p ≤ 0.001

A significant increase in p62 levels in FTLD fibroblasts without the C9-HRE as compared to control fibroblasts was observed (Fig. 2c, g), which might indicate subtle changes in autophagosomal function. p62 forms aggregates and inclusions in the brains of FTLD patients [71]. To examine possible aggregation or changes in p62 subcellular localization, the fibroblasts were stained with a p62 antibody and analyzed by immunofluorescence microscopy. This analysis did not reveal clear cytoplasmic p62 inclusions in FTLD patient fibroblasts. However, quantitative analysis of p62-positive puncta showed a significant increase in the number of puncta in fibroblasts without the C9-HRE (Fig. 3b), in the size of puncta in fibroblasts with and without the C9-HRE (Fig. 3c), and intensity of puncta in fibroblasts with and without the C9-HRE (Fig. 3d) compared to healthy controls, suggesting accumulation of p62 proteins similarly to the Western blot analysis (Fig. 2c, g).

### Fibroblasts from FTLD Patients Respond to Autophagy-Inducing Stimulus Similarly to Control Fibroblasts

**Fig. 4:**
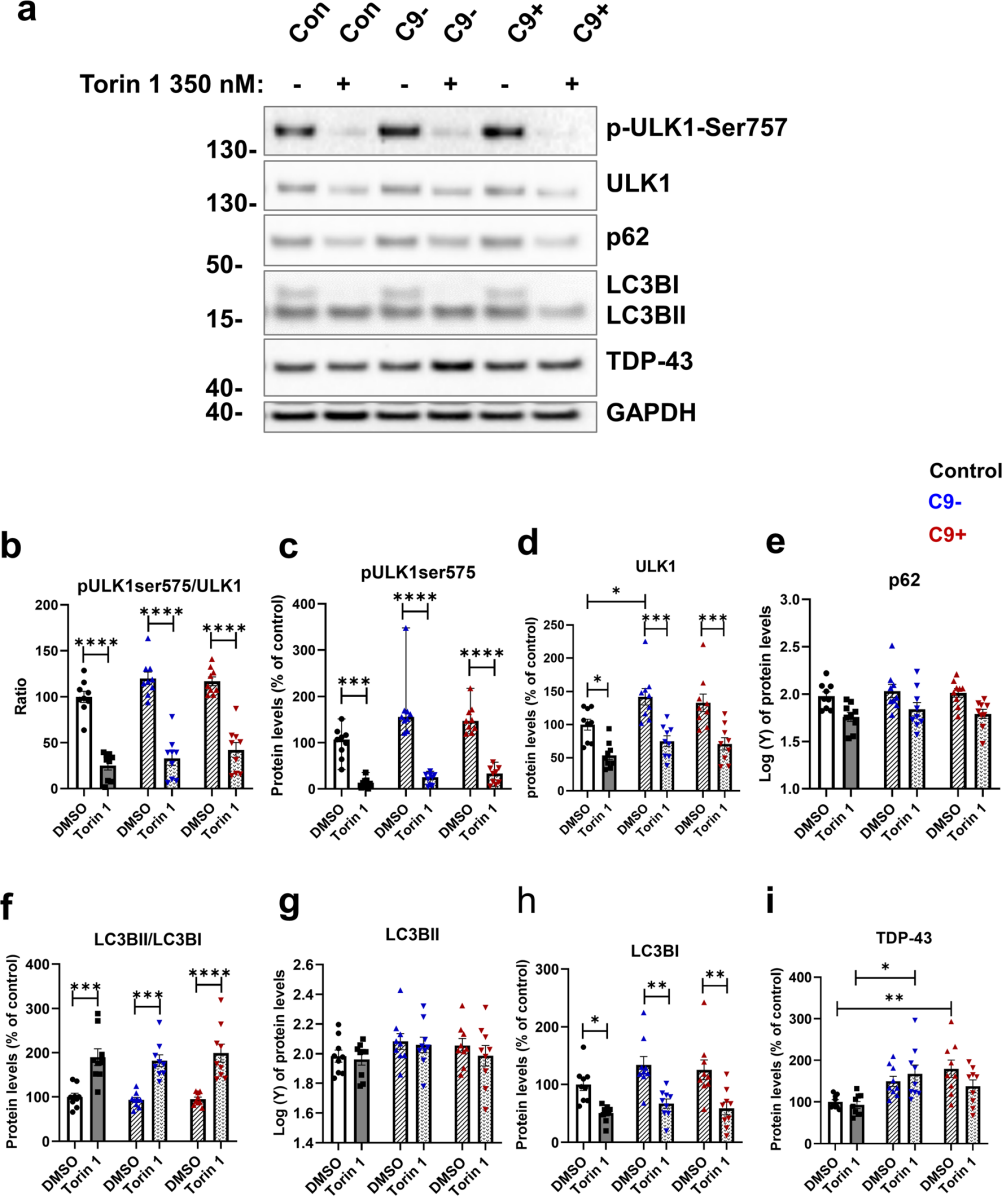
FTLD patient–derived fibroblasts can respond normally to autophagy-inducing stimuli. **a** Representative Western blot images from ULK1, p-ULK1-Ser757, p62, LC3BI and II, TDP-43, and GAPDH from fibroblast cell lysates. Cells were treated with 200 nM Torin 1 overnight to induce autophagy. DMSO was used as a vehicle. **b** Ratio of p-ULK1-Ser757/ULK1. **c** Quantification of p-ULK1-Ser757. **d** Quantification of ULK1. **e** Quantification of p62. **f** Ratio of LC3BII/I. **g** Quantification of LC3BII. **h** Quantification of LC3BI. **i** Quantification of TDP-43. Data are shown as the mean of three biological replicates ± SEM. Two-way ANOVA followed by Tukey’s multiple comparison test was performed for all data sets (**d**–**i**). Data in panels (**e**) and (**g**) were transformed prior to two-way ANOVA. Only *p* values that were significant in the post hoc test are indicated in the graph. *n* = 9 control, *n* = 9 FTLD with C9-HRE, and *n* = 9 FTLD without C9-HRE. *p ≤ 0.05, **p ≤ 0.01; ***p ≤ 0.001, ****p ≤ 0.0001

It has been shown previously that even when basal autophagy is not impaired, pharmacological induction of autophagy can unveil defects in autophagy [28]. We therefore treated the fibroblasts with Torin 1 to induce autophagy and assessed the protein levels of the autophagy-associated proteins ULK1, phospho-ULK1 (p-ULK1-Ser757), LC3BI and II, and p62, as well as TDP-43 (Fig. 4a–i). As expected, treatment with Torin 1 significantly decreased the ratio of p-ULK1-Ser757 to ULK1, indicating induction of autophagy, but no differences could be observed between the fibroblasts from healthy controls and FTLD patients (Fig. 4a, b). Induction of autophagy also significantly increased the LCBII to LC3BI ratio, but again no difference was observed between any of the cells (Fig. 4a, f). These results suggest that fibroblasts from FTLD patients can respond normally to this autophagy-inducing stimulus and that the C9-HRE FTLD fibroblasts do not differ in their response as compared to the fibroblasts from FTLD patients not carrying the C9-HRE nor the control individuals. The levels of TDP-43, another protein showing pathological accumulation in FTLD brain [71, 72], were significantly increased in fibroblasts without the C9-HRE and also showed a trend toward increased levels in fibroblasts with the C9-HRE compared to controls but did not show alterations after induction of autophagy with Torin 1 (Fig. 4a, i).

### Fibroblasts from FTLD Patients Display Unchanged Proteasomal Activity and Subcellular Localization of TDP-43 and Phosphorylated TDP-43 Is Mostly Unchanged

**Fig. 5:**
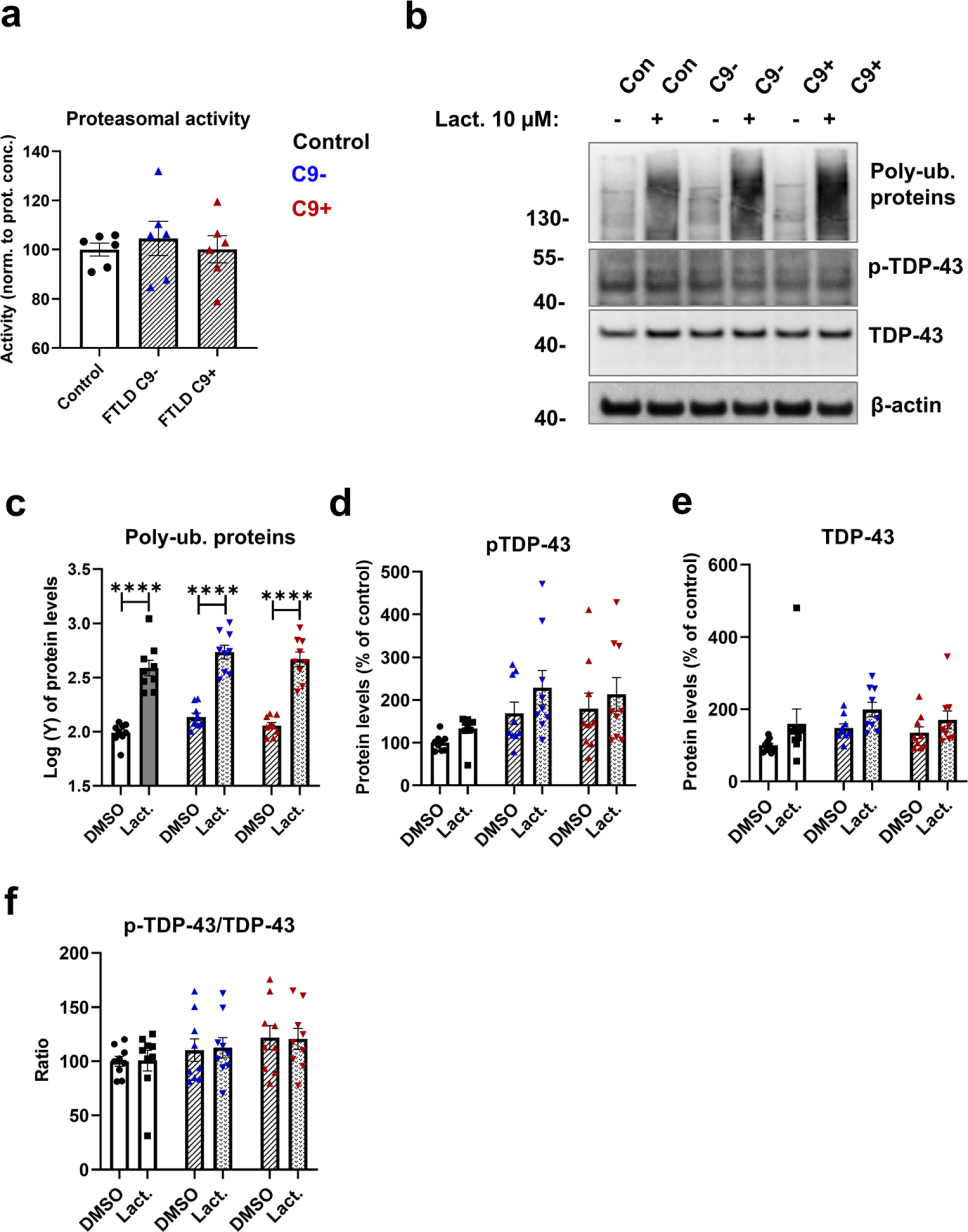
Proteasomal activity is unaffected, and levels of TDP-43 and p-TDP-43 are unchanged by proteasomal inhibition. **a** Proteasomal activity. Data are shown as mean ± SEM. One-way ANOVA followed by Tukey’s multiple comparison test was performed. Only *p* values that were significant in the post hoc test are indicated in the graphs. Data were obtained from two independent experiments. Datapoints represent the average of two or three biological replicates. **b** Representative Western blot images from poly-ubiquitinated proteins, p-TDP-43, TDP-43, and β-actin from fibroblast cell lysates. Cells were treated with 10 μM lactacystin overnight to block protein degradation through the UPS. DMSO was used as a vehicle. **c** Quantification of poly-ubiquitinated proteins. **d** Quantification of p-TDP-43. **e** Quantification of TDP-43. **f** Ratio of p-TDP-43/TDP-43. Two-way ANOVA followed by Tukey’s multiple comparison test was performed. Only *p* values that were significant in the post hoc test are indicated in the graphs. Data in panel (**c**) were transformed prior to two-way ANOVA. *n* = 9 control, *n* = 9 FTLD with C9-HRE, and *n* = 9 FTLD without C9-HRE. ****p ≤ 0.0001

**Fig. 6:**
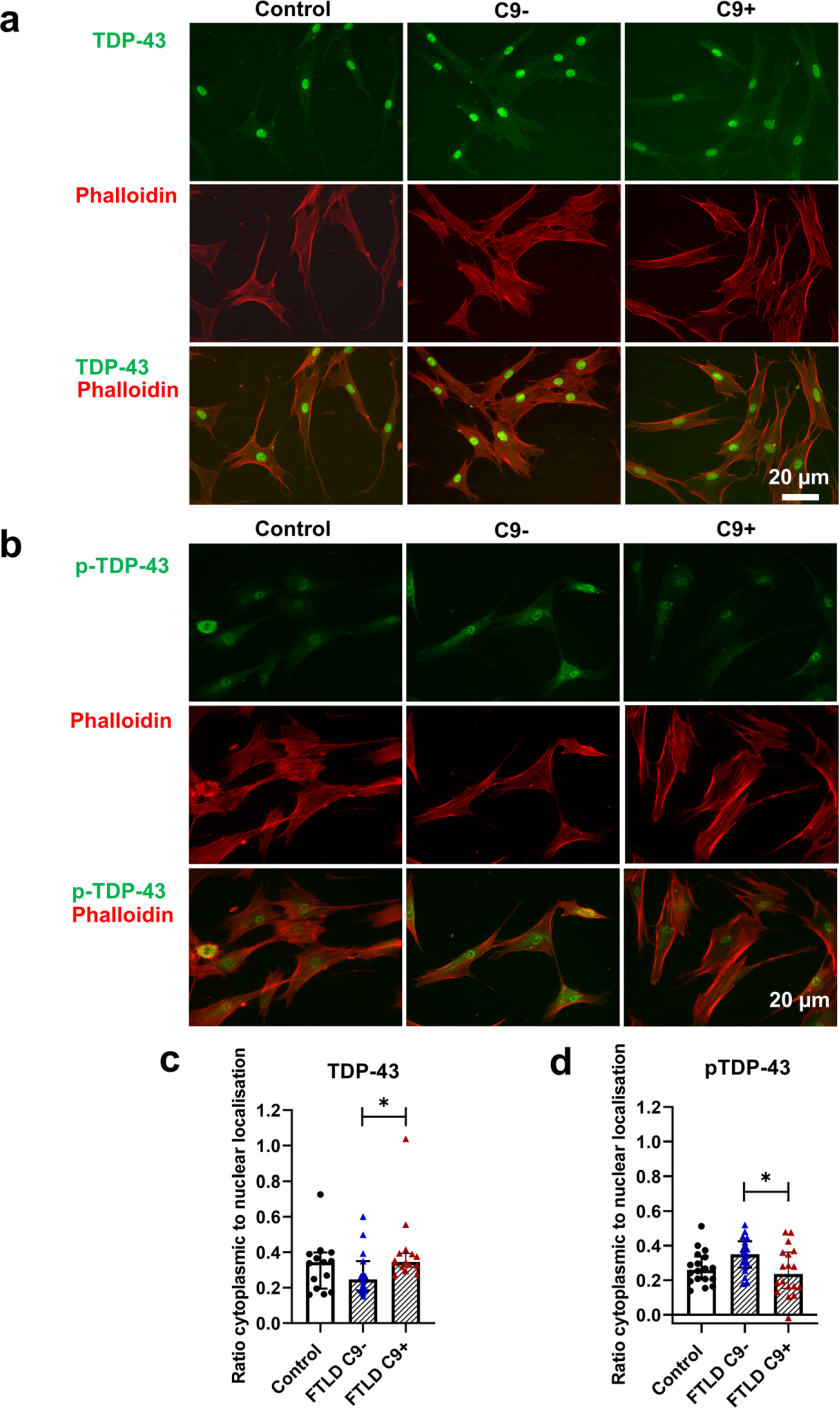
Subcellular localization of TDP-43 and p-TDP-43 are not affected in FTLD patient–derived fibroblasts compared to healthy controls. **a** Representative fluorescence microscopy images of staining with anti-TDP-43 antibody (green) and Phalloidin (red) in fibroblasts of control (left column), FTLD patient without (C9-; middle column), and FTLD patient with the C9-HRE (C9+; right column). **b** Representative fluorescence microscopy images of staining with anti-p-TDP-43 antibody (green) and Phalloidin (red) in fibroblasts of control (left column), FTLD patient without (middle column), and FTLD patient with the C9-HRE (right column). **c** Ratio of cytoplasmic to nuclear localization of TDP-43. **d** Ratio of cytoplasmic to nuclear localization of p-TDP-43. Data are shown as mean ± SEM, and one-way ANOVA followed by Sidak’s multiple comparison test was performed. Number of images analyzed: *n* = 15 for control and *n* = 15 FTLD with C9-HRE and *n* = 15 FTLD without C9-HRE from one experiment. *p ≤ 0.05

In addition to dysfunctional autophagy, defects in the UPS have been suggested to underlie abnormal protein aggregation in neurodegenerative diseases. We next assessed proteasomal activity in control and FTLD patient fibroblasts but did not observe any differences between them (Fig. 5a). Also, blocking the UPS with the proteasomal inhibitor lactacystin led to a similar significant accumulation of poly-ubiquitinated proteins both in healthy control and FTLD patient fibroblasts (Fig. 5b, g). Treatment with lactacystin did not change levels of p-TDP-43 or TDP-43 in either control or FTLD fibroblasts. The RNA-binding protein TDP-43 shuttles between the nucleus and the cytosol [73] and accumulation of cytoplasmic TDP-43 has been observed in the CNS of patients with FTLD and ALS, including C9-HRE carriers, indicating altered subcellular localization [18, 66–68], Examination of TDP-43 subcellular localization demonstrated that TDP-43 is strongly localized in the nucleus in all fibroblasts (Fig. 6a). The cytoplasmic localization was slightly stronger in fibroblasts with the C9-HRE when compared to fibroblasts without the C9-HRE (Fig. 6a, c). p-TDP-43 showed both nuclear and cytoplasmic subcellular localization (Fig. 6b). Fibroblasts without the C9-HRE showed a significantly stronger p-TDP-43 cytoplasmic localization when compared to fibroblasts with the C9-HRE (Fig. 6b, d). However, no significant differences between healthy controls and fibroblasts with or without the C9-HRE were observed.

### FTLD Patient–Derived Fibroblasts Show Significantly Altered Mitochondrial Metabolism

**Fig. 7:**
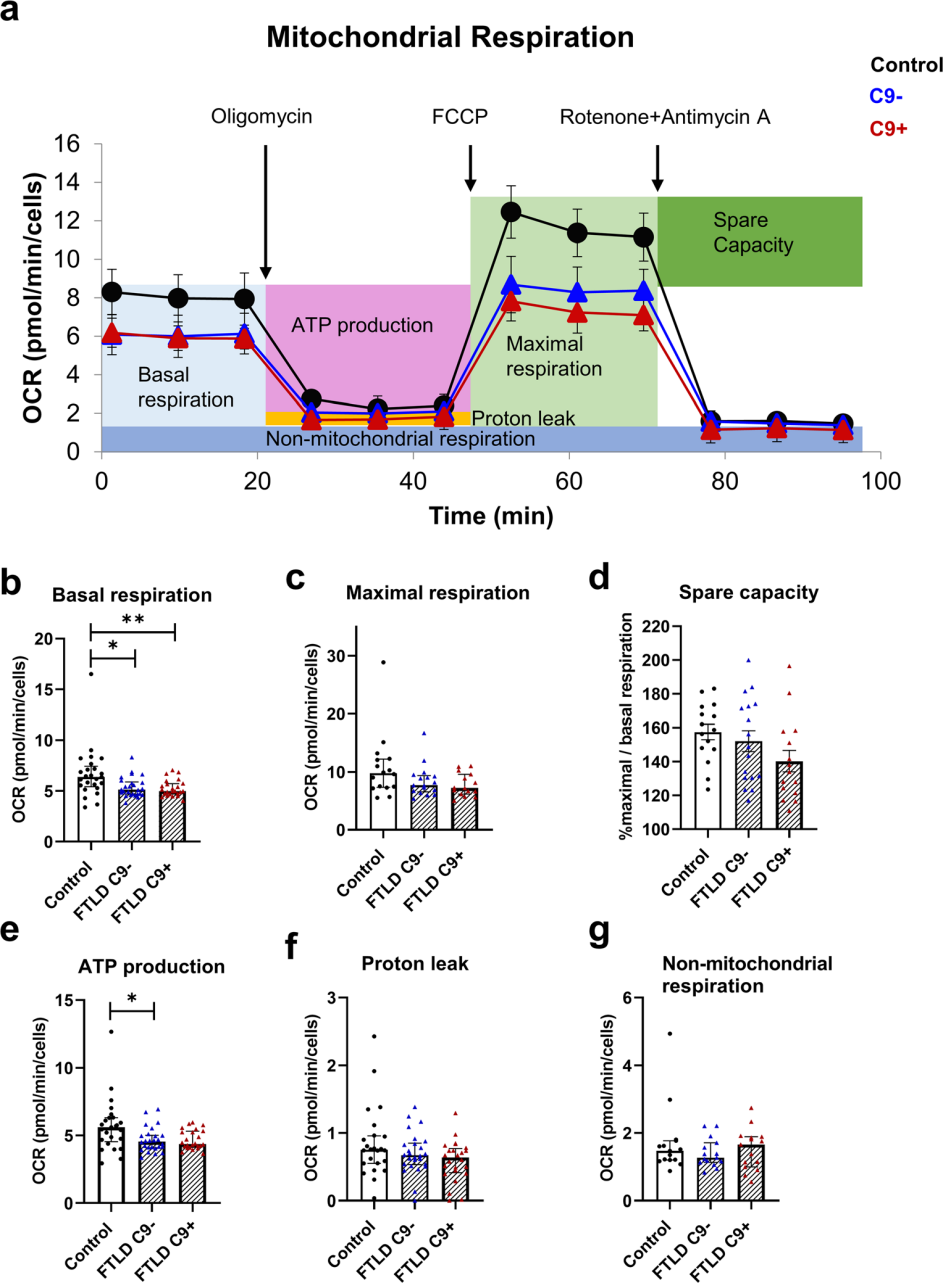
The mitochondrial metabolism of FTLD patient–derived fibroblasts is impaired. Using the Cell Mito Stress Test, several parameters of mitochondrial function were assessed. **a** Example of Cell Mito Stress Test with one control, one C9+, and one C9− fibroblast line. Using the Mito Stress Test assay, several parameters of mitochondrial function were assessed. **b** Quantification of basal respiration. **c** Quantification of maximal respiration. **d** Quantification of spare capacity. **e** Quantification of ATP production. **f** Quantification of proton leak. **g** Quantification of non-mitochondrial respiration. Data are shown as median ± interquartile range and Kruskal–Wallis followed by Dunn’s multiple comparison test was performed (**b**, **c**, **e**, **f**). Data are shown as mean ± SEM, and one-way ANOVA followed by Sidak’s multiple comparison test was performed (**d**). Only *p* values that were significant in the post hoc test are indicated in the graphs. *n* = 15 control, *n* = 15, FTLD with C9-HRE and *n* = 15 FTLD without C9-HRE for maximal respiration and spare capacity and *n* = 24 control, *n* = 24 FTLD with C9-HRE and *n* = 24 FTLD without C9-HRE for other parameters. *p ≤ 0.05; **p ≤ 0.01

A previous study on brain samples of sporadic FTLD patients has shown changes in the expression of several subunits of the complexes of the ETC and significantly reduced enzymatic activity of mitochondrial complexes I, IV, and V [74]. Reduced activity of complexes I, II, III, and IV has also been observed in postmortem spinal cord of sporadic ALS patients [75]. In addition to neurons, mitochondrial dysfunction has been observed in fibroblasts from sporadic ALS cases and ALS or FTLD cases carrying different mutations [60, 61], including patients carrying C9-HRE [62]. To assess mitochondrial function, we examined changes in OCR after injection of different ETC modulators in the control and FTLD fibroblasts (Fig. 7a). A significant reduction in the basal respiration (Fig. 7b) of fibroblasts with and without the C9-HRE was detected compared to controls. Respiration linked to ATP production (Fig. 7e) was significantly reduced in fibroblasts without the C9-HRE and a similar trend (*p* = 0.07) could be observed in fibroblasts with the C9-HRE, suggesting an impaired mitochondrial function. No difference between the fibroblasts of patients with and without the C9-HRE could be observed. OCR related to maximal respiration (Fig. 7c), spare capacity (Fig. 7d), proton leak (Fig. 7f), and non-mitochondrial respiration (Fig. 7g) was similar in FTLD patient fibroblasts and control fibroblasts.

**Fig. 8:**
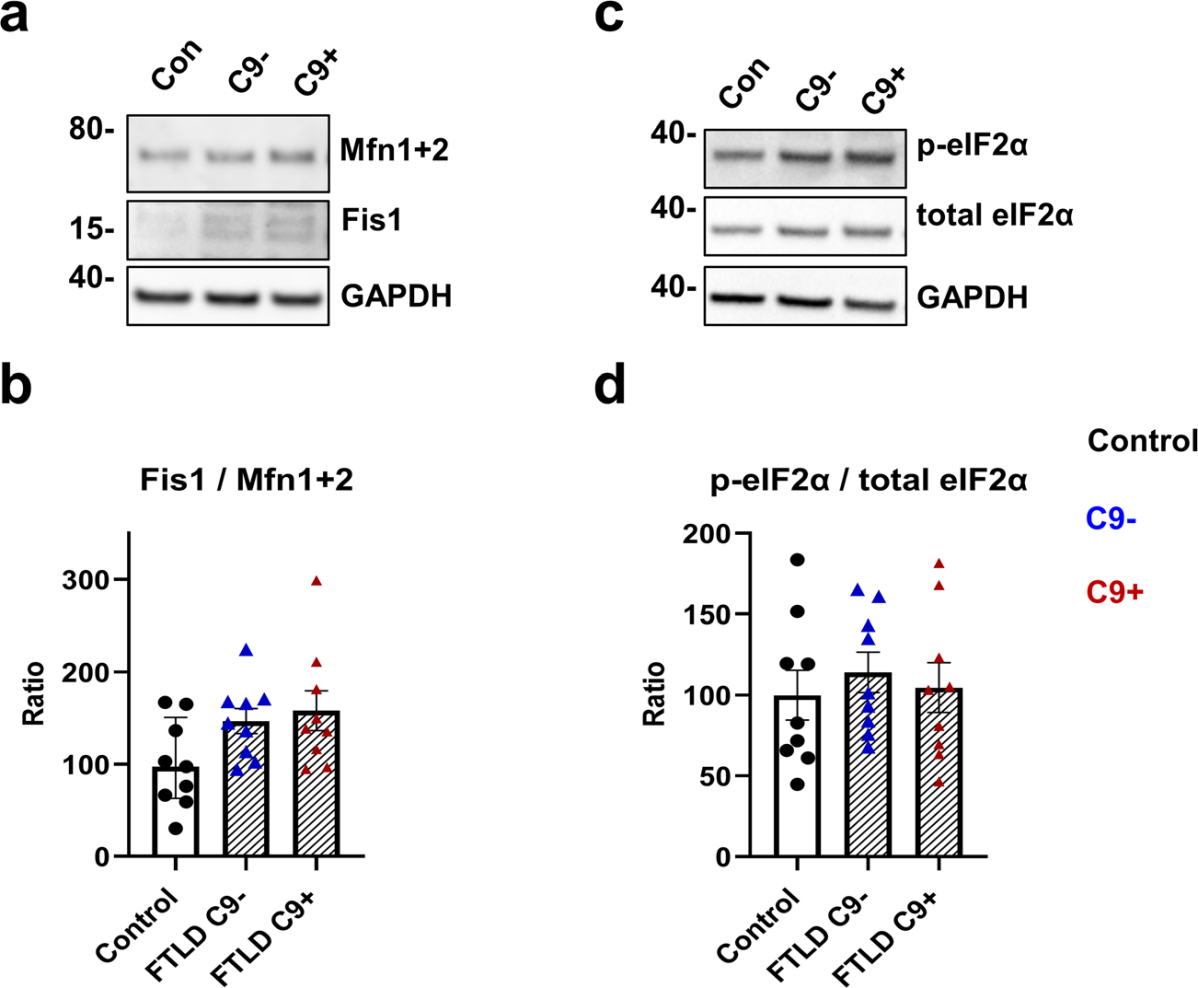
FTLD patient–derived fibroblasts show a trend of increased fission/fusion ratio, but no changes in phosphorylation of eIF2α. **a** Representative Western blot images from Fis1 and Mfn1+2 from fibroblast cell lysates. **b** Ratio of Fis1/ Mfn1+2. **c** Representative Western blot images from p-eIF2α and total eIF2α from fibroblast cell lysates. **d** Ratio of p-eIF2α/total eIF2α. Data are shown as the mean of three biological replicates ± SEM with ratio in controls set to 100. One-way ANOVA followed by Sidak’s multiple comparison test was performed. Only *p* values that were significant in the post hoc test are indicated in the graphs. *n* = 9 control, *n* = 9 FTLD with C9-HRE and *n* = 9 FTLD without C9-HRE

Because deficits in the mitochondrial function were observed (Fig. 7b, e), we next examined the levels of two proteins involved in mitochondrial fusion and fission, Mitofusin (Mfn 1 and 2) and Fission1 (Fis1), to assess potential fragmentation of the mitochondria. A non-significant trend toward increased fission-to-fusion ratio was observed in the FTLD fibroblasts, especially with the C9-HRE (*p* = 0.067) compared to controls (Fig. 8a, b), suggesting that changes in mitochondrial structure might occur in the FTLD patient fibroblasts. Finally, to investigate potential changes upon mitochondrial dysfunction in the interorganelle communication between mitochondria and the endoplasmic reticulum (ER), suggested by previous studies [76, 77], we assessed if increased ER stress could be observed in the FTLD fibroblasts. Attenuation of protein translation through increased phosphorylation of the eukaryotic translation initiation factor 2α subunit (eIF2α) is involved in the unfolded protein response (UPR) associated with ER stress [78]. No differences between the FTLD and control fibroblasts in the ratio of phospho-eIF2α (p-eIF2α) to total eIF2α were found (Fig. 8c, d), indicating that the FTLD patient–derived fibroblasts do not show signs of UPR activation and ER stress.

## Discussion

In the present study, we characterized pathological and functional properties of skin fibroblasts derived from FTLD patients either carrying or not the C9-HRE to evaluate the potential usability of these peripheral and easily accessible cells as platforms in biomarker discovery as well as in drug research.

One of the proposed mechanisms underlying C9-HRE-associated pathogenesis is haploinsufficiency, leading to decreased *C9orf72* mRNA and protein expression. Decreased levels of *C9orf72* mRNA and proteins have previously been detected in the affected CNS areas of the C9-HRE carriers as well as in some peripheral tissues, such as in blood lymphocytes [9, 10, 15]. Our study did not indicate decreased *C9orf72* levels at either mRNA or protein level in the fibroblasts of the C9-HRE carriers, suggesting that the fibroblasts of the carriers do not display signs of haploinsufficiency. In fact, one of the C9-HRE carriers showed elevated *C9orf72* mRNA levels compared to the healthy controls and non-carrying FTLD patients, but this was not accompanied by a rise in the C9orf72 protein isoform A levels. The reason for the increased mRNA levels in this one C9-HRE carrier remains thus far unknown. As the sample size in our study is quite small and the observed difference could reflect natural variation between the individuals, a larger sample set may be needed to further validate the present finding. Intermediate repeat carriers have been reported to show increased *C9orf72* expression in the brain tissue [79] and a recent report [80] showed that *C9orf72* levels in blood were increased in a subset of patients even though the repeat size was larger than the intermediate repeat size of 30 repeats. Therefore, our results together with those by others [79, 80] highlight the complex relationship between *C9orf72* expression and the length of the C9-HRE.

Examination of the fibroblasts from the C9-HRE carriers revealed the presence of nuclear RNA foci, one of the gain-of-toxic-function hallmarks of the C9-HRE. However, the C9-HRE carrier fibroblasts did not display DPR proteins, which are another typical C9-HRE-associated gain-of-toxic-function pathological hallmark. DPR proteins have previously been detected mostly in the neurons of the patients and to a much lesser extent in other brain cells, such as glia. They have not been reported to be present in peripheral tissues, such as blood cells or fibroblasts [65]. Our finding is in line with these studies. In contrast, however, a recent study indicated that specific DPR proteins (poly-GA and poly-GP) could be detected in skeletal muscle samples of a subset of C9-HRE-carrying ALS patients. In addition, in particular poly-GP DPR proteins have been detected in the patient cerebrospinal fluid and therefore suggested to show potential as a biomarker for C9-HRE carriers [81].

Several studies, including ours, have suggested that the C9of72 protein isoform A regulates autophagy, even though it remains controversial based on studies in different cell types if the reduction of C9orf72 protein levels leads to increased or decreased autophagy [16, 27–31, 39–42]. Here, we did not observe any changes in the basal or induced autophagy in the fibroblasts of the C9-HRE carriers as compared to those from non-carrying FTLD patients or healthy controls. This is in accordance with the finding that there were no significant changes in C9orf72 protein levels in the C9-HRE carrier fibroblasts. Moreover, unaltered proteasomal activity and the similar levels of poly-ubiquitinated proteins upon proteasomal inhibition using lactacystin in FTLD patient-derived fibroblasts as compared to healthy control fibroblasts or between the C9-HRE-carrying and non-carrying fibroblasts indicated no changes in the UPS function. In the C9-HRE-carrying fibroblasts, these findings agree well with the fact that they were found to lack the DPR proteins, which in some other studies have been shown to interfere with the function of autophagy and the UPS [44, 45]. On the other hand, in a previous study in sporadic and C9-HRE-associated ALS/FTLD and ALS fibroblasts, proteasomal inhibition with MG132 led to increased levels of poly-ubiquitinated proteins in healthy control but not patient-derived fibroblasts, suggesting impaired proteasomal function in the patient-derived fibroblasts [21]. However, the discrepant findings between these two studies could be due to the use of different pharmacological compounds and treatment conditions to inhibit the proteasomal activity. An increase in p62 levels in the FTLD patient–derived fibroblasts was observed in the present study and this was accompanied by increased number, size, and intensity of p62-positive puncta in the fibroblasts of both C9-HRE carriers and non-carriers. p62 accumulates in different brain regions of ALS and FTLD patients [18] and increased levels of p62 have been observed in fibroblasts from C9-HRE carriers [30]. p62 accumulation can be caused by impaired autophagy or proteasomal activity, but its levels can also change independent of these due to increased transcriptional activation [82–84]. Moreover, in iPSC-derived motor neurons from patients with ALS and FTLD, an increase of p62 was observed without evident changes in LC3-II/LC3-I turnover, further suggesting alternative mechanisms regulating p62 levels [85]. Changes in TDP-43 subcellular localization have been previously described in ALS patient–derived fibroblasts with different mutations, including the C9*orf*72 and *TARDBP* mutations [86]. In that study, cytoplasmic localization of TDP-43 and presence of p-TDP-43 could only be observed in patient-derived but not in healthy control fibroblasts. In sporadic and *C9orf72* ALS/FTLD and ALS fibroblasts [21], significant differences in p-TDP-43 levels and formation of large p-TDP-43 positive inclusions between healthy control and patient-derived fibroblasts were observed only after proteasomal inhibition. In another study on patient-derived fibroblasts carrying *TARDBP* or *UBQLN2* mutations [87], differences in the localization of TDP-43 were also only observed after inhibition of UPS function or induction of oxidative stress. Therefore, marked differences in TDP-43 and/or p-TDP-43 localization or aggregate formation may only become apparent under proteostatic stress conditions. In the present study, a slight increase in levels of TDP-43 could be observed in fibroblasts from FTLD patients, but we did not observe drastic differences in the subcellular localization of TDP-43 or p-TDP-43 between the fibroblasts from FTLD patients in comparison to control fibroblasts under normal conditions. Furthermore, we did not detect signs of TDP-43 or p-TDP-43 aggregation nor intracellular inclusions under proteasomal inhibition. We used lactacystin to inhibit the proteasome, whereas MG132 was used in both previous studies. Thus, it is possible that the different outcomes in these studies are due to the use of different inhibitors to block the proteasomal activity.

Mitochondrial dysfunction has previously been observed in fibroblasts of sporadic ALS cases as well as fibroblasts from patients carrying different mutations, such as *VCP*, *SOD1*, *TARDBP*, or the C9-HRE [59–62]. A study using fibroblasts from three ALS patients carrying a *TARDBP* mutation and three ALS and one FTLD patient carrying the C9-HRE showed changes in mitochondrial function and mitochondrial morphology accompanied by a fragmented mitochondria network in the *TARDBP* mutation-carrying fibroblasts under conditions where glucose in the media was replaced by galactose to switch the mitochondrial metabolism from glycolysis to oxidative metabolism [62]. In the present study, we detected significantly altered mitochondrial function in both C9-HRE-carrying and non-carrying FTLD fibroblasts as compared to healthy control cells, as indicated by the reduced basal respiration and reduced respiration linked to ATP production under standard conditions in glucose-containing medium. These studies altogether suggest that mitochondrial dysfunction is a common characteristic of ALS and FTLD patient fibroblasts.

Morphologically abnormal and fragmented mitochondria were one of the first changes observed in motor neurons of ALS patients [88, 89] and can also be found in animal models of ALS [90]. Subtle fragmentation of the mitochondrial network has also been observed in the fibroblasts of ALS and FTLD patients carrying mutations in *TARDBP* (p.A382T) or the C9-HRE [62]. The functional changes in the mitochondrial respiration in our study were accompanied by a trend toward increased fission-to-fusion ratio, indicating enhanced fragmentation of mitochondria. This finding deserves further examination using alternative methods (e.g., cellular imaging) and different conditions, such as switch of the mitochondrial metabolism route similarly to the previous study [62] or some other form of cellular stress. Fragmentation of mitochondria can impair their function, as shown in a study on ALS patient–derived fibroblasts carrying the *SOD1* mutation. The fibroblasts showed mitochondrial fragmentation and dysfunction as well as inhibition of the Drp1/Fis1 interaction. Reducing mitochondrial fission led to a significant decrease in ROS levels and improved mitochondrial function and structure [91]. Interestingly, several genes linked to mitochondria were deregulated and the enzymatic activity of mitochondrial complexes I, IV, and V were significantly reduced in the frontal cortex of patients with sporadic FTLD [74], further indicating that FTLD pathogenesis may essentially involve dysregulated mitochondrial function.

Mitochondrial dysfunction can also cause ER stress due to ATP depletion or upon disruption of the mitochondrial respiratory chain by nitric oxide, rotenone, or antimycin A [92–95]. A study on fibroblasts from patients with a mitochondrial complex I deficiency, leading to reduced basal respiration and ATP production, showed impaired ER–mitochondria communication [76] and mitochondrial energy dysfunction. Subsequent disruption of inter-organelle communication has been previously shown to induce ER stress [77]. Conversely, ER stress can induce mitochondrial dysfunction [96]. Increased ER stress has been observed in iPSC-derived motor neurons from C9-HRE-carrying ALS/FTLD patients [85]. Here, no change in the phosphorylation of eIF2α, which is phosphorylated by PKR-like ER-localized eIF2α kinase (PERK), one of the kinases activated during the ER stress response, in FTLD patient–derived fibroblasts was detected, suggesting that the observed mitochondrial dysfunction did not induce ER stress in FTLD patient–derived fibroblasts.

## Conclusions

The present data altogether show that the FTLD patient–derived fibroblasts from C9-HRE carriers contain nuclear RNA foci but do not display haploinsufficiency nor express the DPR proteins. Moreover, the C9-HRE-carrying and non-carrying FTLD patient fibroblasts display unaltered UPS function and basal autophagy and can respond to an autophagy-inducing stimulus in a similar manner to the healthy control fibroblasts. Despite unaltered functions of the protein degradation pathways, an accumulation of p62, possibly due to increased transcriptional activation, was evident in both C9-HRE-carrying and non-carrying FTLD patient–derived fibroblasts, suggesting that it represents a common pathological feature in FTLD fibroblasts. However, elucidation of the underlying mechanism of p62 accumulation and its potential, e.g., as a possible biomarker, needs further study. The main pathological finding in this study, which was common to both FTLD patient–derived fibroblasts with and without the C9-HRE, was the marked mitochondrial dysfunction associated with potentially increased mitochondrial fragmentation. Evidence for mitochondrial fragmentation in ALS patient–derived motoneurons as well as deregulated expression of mitochondrial genes in the frontal cortex of FTLD patients have previously been observed. Therefore, balancing mitochondrial function might be an interesting therapeutic target for future studies. The present findings also suggest that patient-derived fibroblasts might represent suitable platforms for biomarker discovery and screening of specific drug effects, such as those affecting mitochondria, in FTLD and ALS research.

## Supplementary Information

Fig. S1 Fibroblasts do not show RNA foci when FISH is performed with the TYE 563-(CAG)6 negative control probe. Representative images of fibroblasts of a C9-HRE carrier (C9+; lower images) and a non-carrier ((C9-; upper images). No RNA foci could be observed when the TYE 563-(CAG)6 negative control probe was used instead of the LNA probe TYE 563-(CCCCGG)3 (PNG 572 kb)

## Abbreviations

2R: Control construct containing the GGGGCC hexanucleotide repeat sequence two times
66R: Pathological construct containing the GGGGCC hexanucleotide repeat sequence 66 times
ALS: Amyotrophic lateral sclerosis
ATP: Adenosine triphosphate
ATP5FA1: ATP synthase F1 subunit alpha
BafA1: Bafilomycin A1
BSA: Bovine serum albumin
C9-HRE: Hexanucleotide repeat expansion in the *C9orf72* gene
*C9orf72*: Chromosome 9 open reading frame 72 gene
CNS: Central nervous system
DAPI: 4′,6-Diamidino-2-phenylindole
DENN: Differentially expressed in normal and neoplastic cells
DEPC: Diethyl pyrocarbonate
DMSO: Dimethyl sulfoxide
DPR: Dipeptide repeat
ECL: Enhanced chemiluminescence
eIF2α: Eukaryotic translation initiation factor 2 α subunit
ER: Endoplasmic reticulum
ETC: Electron transport chain
FCCP: Carbonyl cyanide-4-(trifluoromethoxy)phenylhydrazone
Fis1: Fission1
FISH: Fluorescence in situ hybridization
FTLD: Frontotemporal lobar degeneration
GA: Glycine-alanine
GAPDH: Glyceraldehyde 3-phosphate dehydrogenase
GP: Glycine-proline
GR: Glycine-arginine
*GRN*: Granulin gene
IMDM: Iscove’s modified Dulbecco’s medium
iPSC: Induced pluripotent stem cells
LC3B: Microtubule-associated protein 1 light chain 3B
LNA: Locked nucleic acid
MAPT: Microtubule-associated protein tau
Mfn: Mitofusin
mtDNA: Mitochondrial DNA
OCR: Oxygen consumption rate
p62: Sequestosome-1/ubiquitin-binding protein p62
PA: Proline-alanine
PCR: Polymerase chain reaction
PERK: PKR-like ER-localized eIF2α kinase
PFA: Paraformaldehyde
PR: Proline-arginine
PVDF: Polyvinylidene fluoride
RAN: Repeat-associated non-AUG
ROS: Reactive oxygen species
*SOD1*: Superoxide dismutase 1 gene
*TARDBP*: TAR DNA binding protein gene
TBST: Tris-buffered saline with 0.1% Tween 20
TDP-43: TAR DNA-binding protein-43
*UBQLN2*: Ubiquilin-2 gene
UPR: Unfolded protein response
UPS: Ubiquitin–proteasome system
*VCP*: Valosin-containing protein gene
