# Supplementary figures and images for: FTLD Patient–Derived Fibroblasts Show Defective Mitochondrial Function and Accumulation of p62
Source: Mol Neurobiol. 2021 Jul 30;58(11):5438–58. doi: 10.1007/s12035-021-02475-x (PMC8599259; doi:10.1007/s12035-021-02475-x)

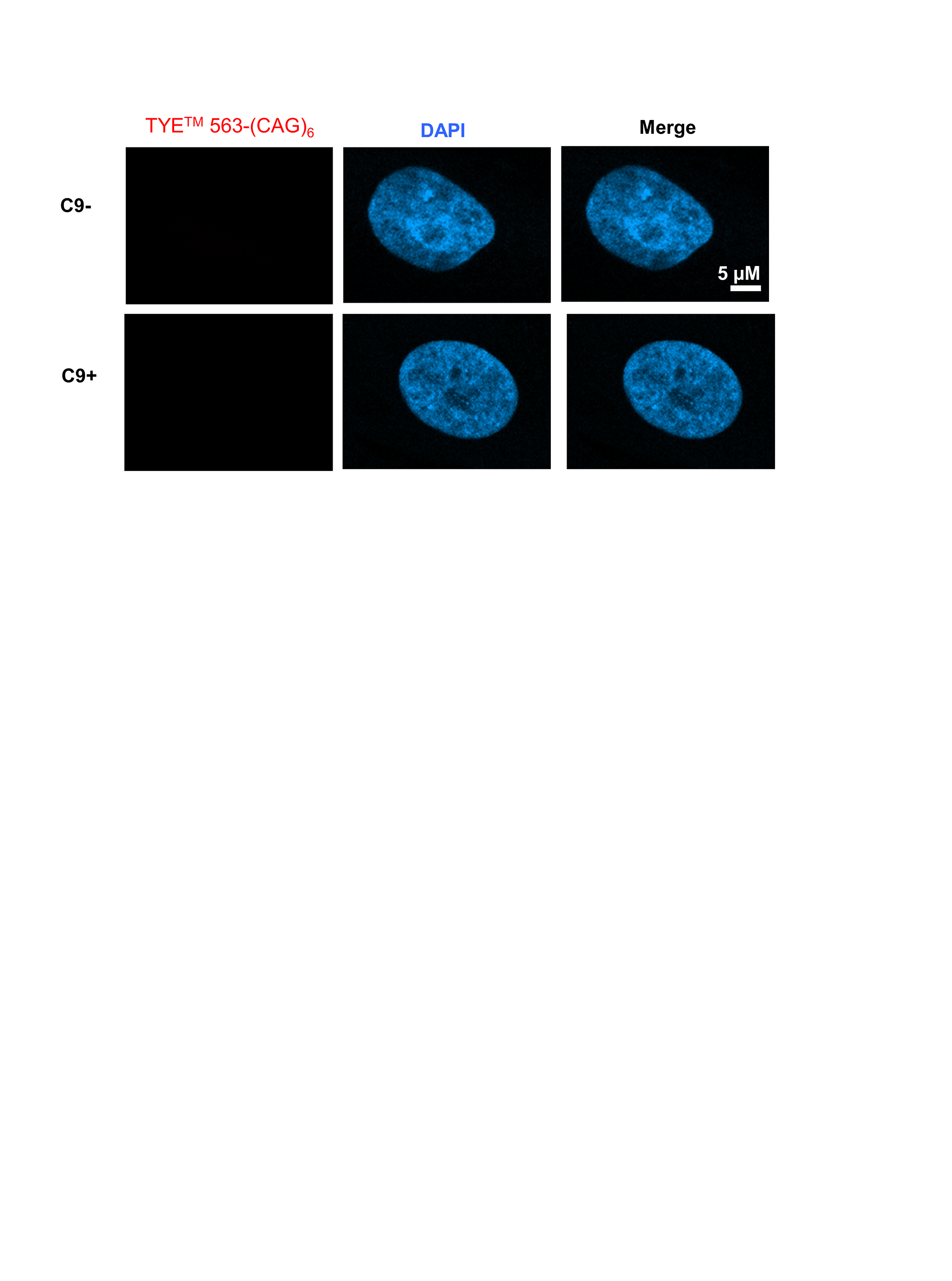

Supplement: Supplementary file 1 — Fibroblasts do not show RNA foci when FISH is performed with the TYE 563-(CAG)6 negative control probe. Representative images of fibroblasts of a C9-HRE carrier (C9+; lower images) and a non-carrier ((C9-; upper images). No RNA foci could be observed when the TYE 563-(CAG)6 negative control probe was used instead of the LNA probe TYE 563-(CCCCGG)3 (PNG 572 kb) [file 12035_2021_2475_MOESM1_ESM.png]
